# Can new urbanization and ecological environment achieve synergistic development? Empirical evidence from 63 counties in Zhejiang, China

**DOI:** 10.1371/journal.pone.0291867

**Published:** 2023-09-21

**Authors:** Lindong Ma, Weixiang Xu, Yuanxiao Hong, Shouchao He, Chenjun Liu, Qian Ning

**Affiliations:** 1 School of Management, Zhejiang University of Technology, Hangzhou, 310023, China; 2 Xingzhi College, Zhejiang Normal University, Jinhua, 321100, China; 3 School of Economics and Management, Wenzhou University of Technology, Wenzhou, 325027, China; 4 Zhijiang College, Zhejiang University of Technology, Shaoxing, 312030, China; 5 College of Humanities, Zhejiang Normal University, Jinhua, 321000, China; Nanjing Audit University, CHINA

## Abstract

As China’s urbanization accelerates, ecological environmental issues have become increasingly prominent, and how to achieve the synergistic development of urbanization and ecological environment is worth exploring. The paper uses the Super-SBM model and the improved entropy method to calculate the ecological efficiency and the new urbanization in 63 counties in Zhejiang Province from 2000 to 2019. Furthermore, the coupling coordination degree between new urbanization and ecological efficiency is discussed with the coupling degree model, Markov chain, and spatial correlation methods, and its influencing factors are explored by the geographic detector. The results show that: (1) The development trends of new urbanization and ecological efficiency in Zhejiang Province counties both present a "U" shape. Their inflection points appeared in 2005 and 2006, respectively. The gap between counties is gradually narrowing. (2) The coupling coordination degree between new urbanization and ecological efficiency in Zhejiang Province counties also develops in a "U" shape with the minimum value appearing in 2006. Its temporal evolution is dominated by advancement towards a higher level and maintenance of the original type, with most countries advancing from General Disorder to Preliminary Coordination. There is a good positive correlation in the spatial distribution, showing significant High-High and Low-Low agglomeration. (3) In detecting the driving factors, the explanatory power of economic development, natural conditions and social conditions diminishes sequentially. The interaction groups mostly are nonlinear enhancements, and the rest are all two-factor enhancements. Social factors are the main interaction objects. (4) The empirical analysis verified the efficacy of the "Two Mountains" theory and the importance of government investment in the regional coordinated development.

## 1. Introduction

Since the reform and opening-up, China’s urbanization has developed rapidly, jumping from 17.9% in 1978 to 63.89% in 2020. However, urbanization is a “double-edged sword” [1]. The urbanization process shows prominent characteristics of "high growth and high pollution" in China [2], which is bound to be accompanied by many influences and changes. First of all, with the acceleration of urbanization, the increase in the urban population will inevitably lead to a rise in energy demand and severe environmental pollution problems [3]. Second, with the development of cities, the expansion of urban areas will have an impact on the regional ecology and even have a more significant impact on people’s production, life, and society, and also bring many problems such as air pollution [4] and noise [5] and so on. Because of this, China’s cities are now facing unprecedented ecological and environmental pressure from the excessive consumption of natural resources, environmental destruction, and pollutant emissions [6]. Of course, urbanization has favorably contributed to economic development, social progress, city-level enhancement, and improving living standards. Therefore, in the process of promoting urbanization, we try to give full play to the positive effects of the ecological environment brought about by urbanization while eliminate the adverse effects, thus beneficially promoting the urban ecological environment and thus contributing to the advancement of urban ecological efficiency. At the same time, the government is vigorously promoting the ecological environment project, which in turn contributes to cities’ development, making the coordinated development between the two systems possible. Thereby, this paper aims to explore if the two systems can achieve coordinated development.

The advancement of urbanization brings challenges to the ecological environment. The United Nations has issued a systematic framework of Sustainable Development Goals (SDGs) from the economic, social, and environmental perspectives [7]. To achieve these SDGs, China has also proposed many strategies [8], but it still has a long way to go [9]. Among them, the most representative is the "Two Mountains" theory proposed by Xi Jinping in Zhejiang in 2005, which has been implemented ever since. This theory believes that "lucid waters and lush mountains are invaluable assets" and strengthens the concept of ecological civilization and green development. It aims to realize the dialectical unity of environmental protection and economic and social development, to coordinate economic and social development with population, resources, and the environment, and to make green mountains create significant ecological, economic, and social advantages. As China’s economic and social development enters a new normal, the urbanization process has entered a new stage. The measurement of urbanization has been upgraded from a single population indicator to a comprehensive one involving population, economy, society, and environment, which is known as the new urbanization in this study. At the same time, regarded as an indicator of ecological development status and development level, the academic community has deeply recognized and studied the ecological efficiency. In the context of this turning point, it is of great theoretical value and practical guiding significance to explore the relationship between new urbanization and ecological efficiency and the influencing factors to accelerate their coordinated development [10], and quantitatively verify whether the "Two Mountains" theory is effective in a further step. This study can provide theoretical support and policy reference for countries and regions with similar situation.

There have been many studies on the relationship between urbanization and ecological efficiency, mainly concentrating on the following aspects: (1) Theoretical research. Fang and Wang (2013) [11] studied the interactive coercing effect of urbanization and ecological efficiency and the risk effect of interactive coercion, providing scientific decision-making basis for the healthy development of urbanization in China. (2) Evaluation model. Wei et al. (2014) [12] released a new comprehensive index system for evaluating urbanization and ecological efficiency with the theory of interactive coercion. Bai et al. (2018) [6] calculated the urbanization and ecological efficiency in different cities in China by the super-DEA model. Yu (2021) [1] measured the ecological effects of new urbanization based on provincial panel data. (3) Unidirectional effects (the effects of new urbanization on ecological efficiency). Yao et al. (2021) [13] analyzed the respective and widely different linear impacts of urbanization (divided into social, demographic, and spatial by its internal structure) on ecological efficiency using the spatial Durbin model based on the panel data of 30 provinces in China from 2008–2017. Ecological efficiency is a complex indicator [14]. There may be a mutual causal possibility of interaction between urbanization and ecological efficiency [15]. The robustness test is not accounted for in the paper, so whether endogeneity exists is questionable. There is also a study [15] based on provincial panel data of China from 2008 to 2019, which concludes that urbanization has a U-shaped effect on ecological efficiency. Besides, China is in the early negative phase, and the turning point has yet to arrive through the Tobit model. It can be found that the article ignores the spatial effects [16] and inter-regional differences that exist in ecological efficiency [17]. (4) Two-way effect (interaction of urbanization and ecological efficiency) and coupling coordination degree model. Liu et al. (2018) [18] used the coupling coordination degree model and spatial autocorrelation to measure the coupling coordination degree between urbanization and the ecological environment in 30 provinces in China. The results show that the coupled coordination degree of the two systems in each province gradually improved, and spatial differences weakened from 2005 to 2015. It predicts that the coordination state of urbanization and the ecological environment is improving. Another study [19] based on the city level has the similar results. Wang et al. (2019) [20] who set a coupling coordination degree model to analyze the coupling effect between energy-environment efficiency and urbanization, found differences in the development of individual urban clusters. Ariken et al. (2021) [10] combined the coupling coordination degree model and GTWR models to analyze the coupling degree and spatial-temporal heterogeneity of urbanization and ecological environment quality in the Silk Road Economic Belt (9 provinces), concluding that there are interactions between urbanization and ecological environment quality, and that there is regional heterogeneity in coupling coordination, with some provinces decreasing while others increasing. In addition, of course, others have explored the coupling coordination between other ecological benefits and new urbanization, such as the coupling coordination between tourism ecological efficiency and new urbanization [21,22] and the interaction between urbanization and ecological total factor energy efficiency [23], etc. From the existing literature, the study on the relationship between new urbanization and the ecological efficiency has been relatively abundant, and some consistent conclusions have been drawn, such as the existence of a spatial correlation in ecological efficiency and the interaction between the two systems. However, studies of new urbanization and ecological efficiency have primarily focused on the provincial level and urban clusters, with no consistent conclusions in the development patterns of the coupling coordination between them at the county level, and the factors influencing the coupling coordination between them have not been investigated.

The following aspects need to be further improved for the existing research on the relationship between new urbanization and ecological efficiency. Firstly, although there are many studies on the coupling coordination between new urbanization and the ecological environment, more consistency is required in the conclusions. There is a gap in the research on coupling coordination between new urbanization and ecological efficiency, which is one of the essential manifestations of the ecological environment system; therefore, studying the coupling relationship between them can fill this gap and enrich their coordinated development. It should be considered that both urbanization and ecological efficiency are comprehensive and complex systems, so each system should have a relatively complete and scientific evaluation index composition. When studying the relationship between the two, we should not use a single indicator as a representative to study one system’s relationship with the other system in isolation. Secondly, in the selection of research scope, the current research is mainly based on the national level (provincial level) [1,10] and regional level (prefecture-level city level) [20]. However, new urbanization and ecological efficiency have heterogeneity in geographic space and particularity in regional development. Due to their unique spatial dependence and the need for regional development consistency, counties are an essential perspective to see the state of social and economic growth [24]. When the decision-making unit is too macro, capturing more micro-level information is impossible [25]. Therefore, an in-depth analysis at the county level is necessary. Thirdly, due to the significant differences across regions and varied natural conditions, in addition to the commonly used methods such as spatial measurement, there are other new methods to explore geographical heterogeneity and find out other factors that may affect the relationship between the two. For example, measurement methods generally follow linearity and avoid collinearity, and have a limited sample size, which makes them ignore some other influencing factors.

**Fig 1:**
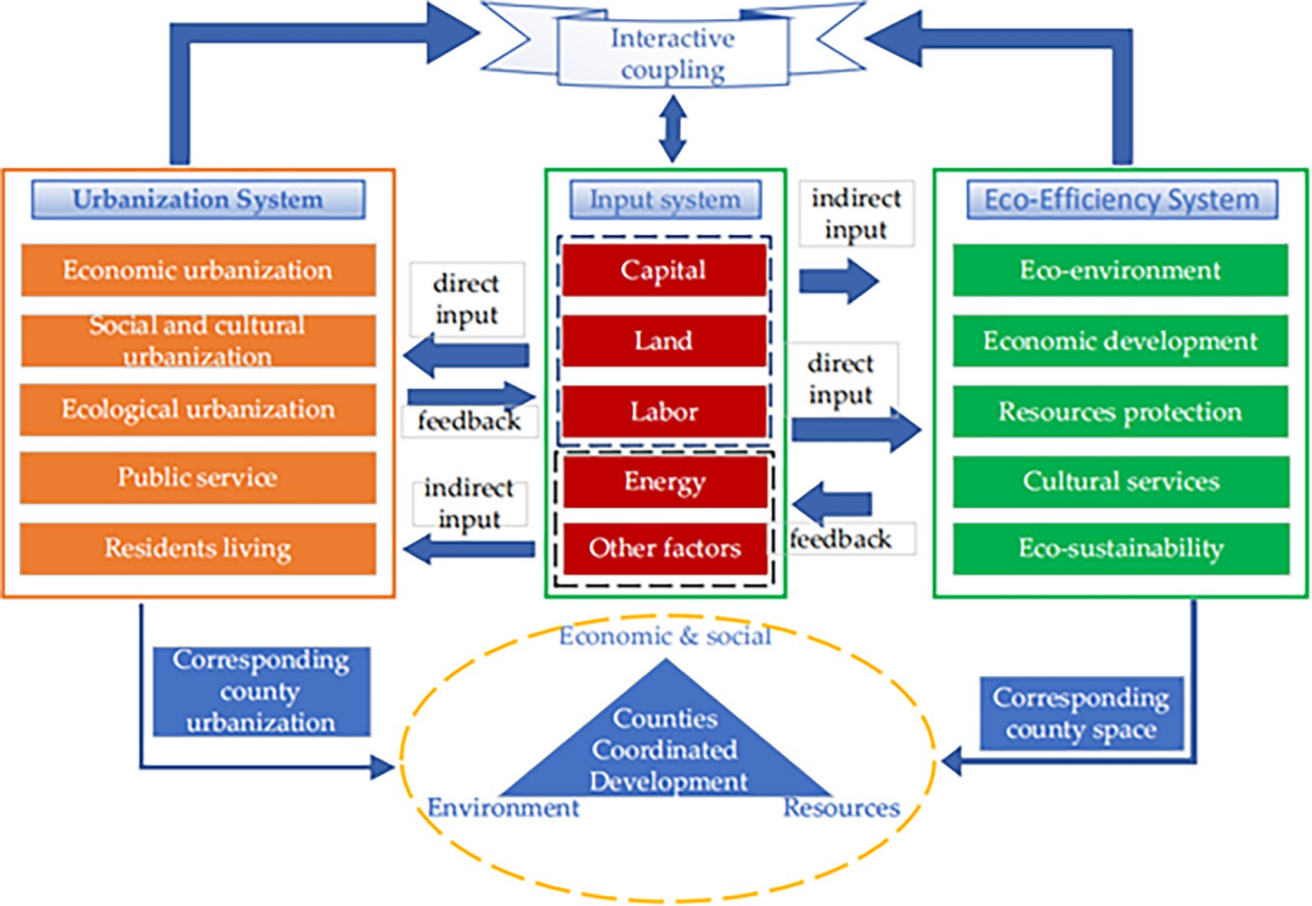
Thesis framework and research flowchart.

To this end, this paper aims to (1) Establish a more scientific index system. Starting from the actual situation of the research area and referring to the previous research, this paper measures the new urbanization from five aspects, including population, economy, society, space, and culture (see Fig 1). Similarly, ecological efficiency is scientifically reflected through five elements as follows: environment, economic development, resource protection, cultural services, and sustainability. (2) To effectively break the phenomenon that various regions are averaged on a macro scale, and objectively reflect the actual situation of each region, following the availability, scientificity, and continuity of official data, we choose to conduct research at the county level in Zhejiang China to fill the research gap in this field. (3) In terms of the research method, the improved entropy method is used to determine the new urbanization level, the Super-SBM model is applied to measure the ecological efficiency, and the coupling coordination model is exploited to study the relationship between the two. Moreover, we use geographic detectors to more comprehensively and scientifically explore the effects of relevant factors on the two systems, avoiding the disadvantages of linearity and multicollinearity in previous measurement methods.

In the rest of the paper, we first conduct a theoretical analysis in Section 2. Then, we present the methods and data sources in Section 3. We report the new urbanization and ecological efficiency results and further analyze their relationship in Section 4. Furthermore, the factors influencing the coupling coordination degree between new urbanization and ecological efficiency in Zhejiang Province counties are elaborated in section 5. Based on the results obtained from the previous analysis, three critical issues raised in this paper are further analyzed and discussed in Section 6. Finally, based on the findings, we provide conclusions and insightful policy implications for promoting the coupling coordinated development of new urbanization and ecological efficiency in Section 7.

## 2. Theoretical analysis

New urbanization is a complex process in which economic structure, social structure, production and lifestyle undergo fundamental changes. It involves various factors, including population, economy, space, society, and culture. Its inherent logic can be understood as follows: demographic urbanization is the core, economic urbanization is the driving force and carrier of urbanization in the regions, spatial urbanization is the representation of the population, cultural urbanization is the intellectual support for the sustainable development of urbanization, and social urbanization presents the changes in people’s lifestyle, behavior habit and value that accompany this process. Ecological efficiency is comprehensively evaluated from five aspects: eco-environment, economic development, resource protection, cultural services, and eco-sustainability. Among them, the ecological environment and economic development indicate the urban ecological environment’s bearing capacity and economic transformation capacity, respectively. Resource protection and cultural services refer to the degree of resource protection and the importance of human beings to the urban ecosystem. At the same time, ecological sustainability reflects the cyclicality and sustainability of the ecological environment in the city.

The relationship between new urbanization and ecological efficiency is a bidirectional interactive coupling [26]. New urbanization mainly establishes links with the ecological efficiency system through population growth, economic development, and spatial expansion. Population urbanization is primarily reflected in the increase of urban population density and consumption level, which would result in increased intensity of human demand from the ecosystem and increased pressure on the urban ecological environment [15]. Economic urbanization is mainly reflected by the changes in production scale and industrial structure, playing a dual role in the ecological environment: on one hand, the expansion of non-agricultural production activities such as industry will increase resource consumption, increasing the threat to the ecological and environmental space [27]; on the other hand, the economic development will bring more investment in environmental protection [28]. With more advanced production methods and clean technologies, the unit consumption of resources and environmental pollution by enterprises can be reduced [29]. At the same time, the protection of the ecological environment will be further improved. Overall, population and economic urbanization have promoted the transformation of urban and rural regional landscapes, that is, spatial urbanization, as evidenced by a rise in urban construction density and geographical scope. This process will directly reduce the area of suitable construction land available, and the disordered spread of construction land will cause dismemberment and encroachment in the ecological space, posing additional threats to the current sustainable development of the ecological environment. The irrational allocation of urban construction land and ecological space will weaken the permeability of ecological elements in the built environment and reduce the virtuous cycle ability of the ecological environment [13]. However, with the enhancement of people’s ecological awareness, reasonable space control has gradually become an effective way to alleviate the harm of urbanization to the ecological environment [30]. Social urbanization has effectively helped various social contradictions and improved people’s welfare, thus further positively effecting the ecological environment construction and its sustainable development.

Ecological efficiency is an indicator value demonstrating the comprehensive and holistic nature of social resource depletion, ecological and environmental conditions, and economic development [31]. As a result, its development is bound to have a positive impact on the promotion of new urbanization. Firstly, the accelerated development of eco-economy further increases green economic efficiency, thus beneficially promoting the green economic development level of urbanization [32], benefiting the sustainable development of the ecological environment, pushing the excellent transformation of the ecological environment, further optimizing the spatial layout of economic development of urbanization and its development process, thus enabling the benign development of new urbanization [33]; Secondly, the improvement of ecological efficiency is highlighted by the apparent decrease of pollutant emissions, the slowing down of the pressure on the ecological environment, and the reduction of ecological resource consumption [1,6]. At the same time, it has well promoted the transformation of the regional environment and the development of the new urbanization [34]. The improvement of social resource protection and the strengthening of the radiation of the benign ecological culture has established an excellent soft environment for the creation and better implementation of resource conservation and a peaceful environment for the whole society. It not only makes the social resources more fully protected and used, but also creates an excellent social habit for the operation and better implementation of a conservation-oriented society and an eco-friendly social governance system. In sum, the improvement of ecological efficiency sets a good benchmark for realizing the soft environment of the new urbanization, ultimately promoting the further development of the new urbanization.

The above analysis shows an inevitable overlap between regional ecological efficiency and new urbanization, but they have different focuses. The development of ecological efficiency promotes the optimization of the material space and time process of urbanization, thus promoting the benign development of new urbanization [35], and the result of urbanization also beneficially promotes the improvement of ecological efficiency, so there is a coupling between the two. New urbanization corresponds to the urban space [36]. In contrast, ecological efficiency corresponds to the county space, and these two aspects show a correlation of "point and surface, source and flow" in urban and county areas. Whether it is the positive effects caused by the circle radiation on population economic welfare or the adverse effects caused by the disorderly discharge of industrial wastewater and waste gas, the vast county areas have become a spatial carrier that carries the positive and negative impacts of various outputs. In contrast, the urban areas have become the ultimate beneficiary [37]. Therefore, a high coupling relationship between the two systems is an essential symbol of the healthy development of the regions. The essence of coupling is to bridge the "heterogeneity" of human intervention and the "origin" of nature, and the coupling relationship between ecological efficiency and urbanization is embodied in the spatiotemporal process of "mutual adaptation" between urban development and natural production.

## 3 Materials and methods

### 3.1 Study area

**Fig 2:**
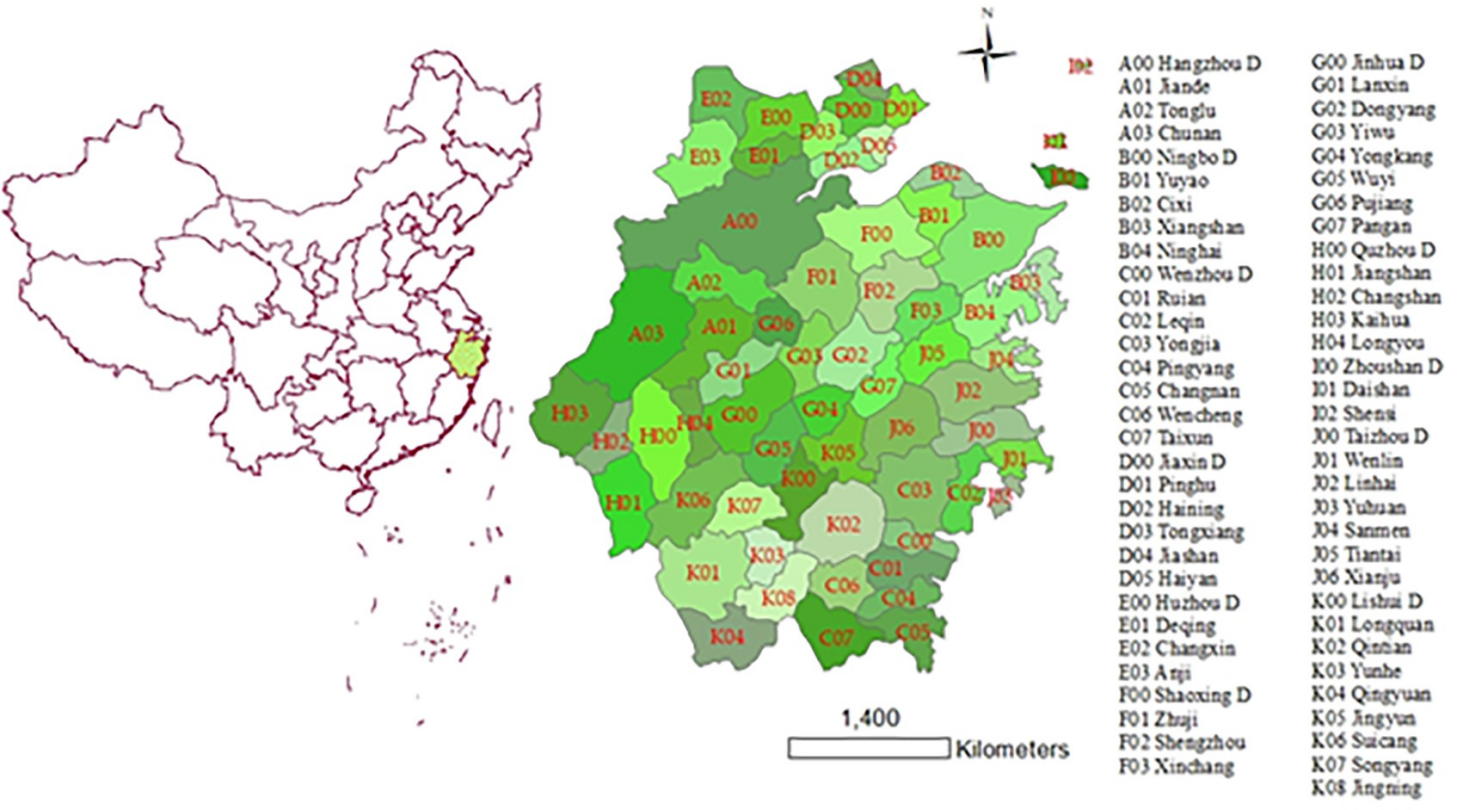
Zhejiang’s location and 63 counties. The map is obtained according to the map approval number: Zhejiang S(2022)34 https://zhejiang.tianditu.gov.cn/standard/view/842c093084084add9788a14a36536525, which complies with the appropriate terms of service https://zhejiang.tianditu.gov.cn/about/clause.

Regarding selecting research objects, the panel data of 63 counties in Zhejiang Province from 2000 to 2019 are selected. Zhejiang Province (118°01’~123°10’E, 27°02’~31°11’N) is located on the southeast coast and the south wing of the Yangtze River Delta. Mountains and hills dominate the terrain, and there is a saying that "seven mountains, one water, and two fields.” By the end of 2019, the permanent resident population of Zhejiang was 58.5 million, and the urbanization rate reached 70%, an increase of 21.33% over the past 20 years. However, the gap between counties is large, such as Yuyao, Yiwu, and Cixi urbanization rate has exceeded 80%, while Wencheng, Taishun, and Chun’an are below 58%. Since 2005, Comrade Xi Jinping put forward the "two mountains" theory that "lucid waters and lush mountains are invaluable assets. "Zhejiang has comprehensively launched four battles of blue sky, clear water, pure land, and clean waste. It has won the national "atmospheric ten" and "water ten" assessments excellently, and 50 county-level cities have built clean air demonstration zones. Through the "five-water co-governance" and the completion of the task of eliminating inferior V. water quality sections three years ahead of schedule, the number of national-level ecological civilization construction demonstration counties and the practice and innovation base of " lucid waters and lush mountains are invaluable assets " ranks first in China. We take the administrative divisions in 2018 as the base map (Fig 2) and use ArcGIS 10.6 for spatial analysis.

### 3.2 New urbanization index system

In line with the general definition of urbanization and the trajectory for China’s urbanization set out in the National New Urbanization Plan (2014–2020) [20], the new urbanization system is a complex and comprehensive indicator system, which is very different from a traditional one which is only measured by the urban population [38]. This paper has referred to the literature in recent years [6,10,38–44] and the new type of urbanization evaluation index system of the quality city (GB/T 39497–2020), which mainly focuses on demographic, economic, social, and spatial aspects. However, regarding the people-centered essence and the need for civilization construction and development in the new urbanization, the role and influence of the cultural atmosphere on regional urbanization construction should not be underestimated, as the regional cultural atmosphere will affect all aspects of life and work. To this end, this paper adds an evaluation indicator of cultural urbanization based on the original four hands to further improve the pertinence, scientific, and integrity of the new urbanization measurement system. Under five first-level hands, each indicator comprises three variables to ensure a more comprehensive and objective reflection of the facts for more specific information about the indicators (see Table 1).

**Table 1:** New urbanization comprehensive evaluation index system.

First-level index	Second-level index	Variables & Direction	Unit
Economic urbanization	Development of mass effect	U1: Per capita GDP (+)	CNY
		U2: Ratio of general public budget revenue to GDP (+)	%
	Economic structure	U3: The percentage of the total value of the second and tertiary industry in GDP (+)	%
		U4: The proportion of the non-agricultural population (+)	%
	Innovation capability	U5: R&D expenditure as a percentage of GDP (+)	%
		U6: Proportion of high-tech enterprises in the total number of registered enterprises (+)	%
Social and Cultural Urbanization	Social governance	U7: urban registered unemployment rate (-)	%
	Cultural development	U8: Ratio of teachers to students in compulsory education (+)	%
		U9: The number of books per 10^4^ people (+)	Books
Ecological environment	Ecological protection	U10: Green coverage rate of urban built-up areas (+)	%
	Environmental Quality	U11: Average annual PM2.5 (-)	Mg/m^3^
	Governance effect	U12: Comprehensive utilization rate of industrial solid wastes (+)	%
		U13: Rate of centralized sewage treatment (+)	%
Public service	Infrastructure	U14: The proportion of public service expenditure in general budget expenditure (+)	%
		U15: Urban central water supply drinking water quantity up to standard rate (+)	%
	Public security	U16: Death rate of production safety accidents per GDP of 100 million yuan (-)	%
		U17: Per 10^4^persons who committed a criminal offense and were sentenced to death (-)	%
	Business environment	U18: The ratio of total imports and exports to GDP (+)	%
Residents living	Living standard	U19: Per capita disposable income to per capita GDP ratio (+)	%
	The well-being of the people	U20: Number of beds per 100,000 persons (+)	Beds/10^5^ persons

### 3.3 Ecological efficiency index system

As an essential sign of sustainable development, ecological efficiency has been paid attention to by both society and scholars [45]. It plays a vital role in the coordinated development of the environment and culture [46]. Before evaluating the ecological efficiency in urban areas, we need to build a corresponding input and output index system. On the premise of referring to the existing relatively mature ecological efficiency index systems [47,48], this paper selects water resources, energy, labor, land, and capital as inputs (see Table 2). Specifically, water resources are an essential part of ecological resources and play an irreplaceable role in both production and living and ecological sustainability, so the amount of water used by the whole society is calculated as an input variable; energy, as an essential component to maintain the operation of organization, is replaced by the amount of electricity used by the whole society, as it is considered that Zhejiang Province is mainly consumed by electricity; labor is the creation of wealth, it also has a decisive influence on the ecological environment, and is usually measured by workers, expressed by the number of urban workers; land resources are the carrier and means of production for the development of sustainable human production and life in the ecological environment, so it is expressed by the area of urban construction land; at last, it’s the capital, as the most important means of production and a measure of the accumulation of ecological environment construction and production and life, this input is expressed by the capital stock. Goldsmith’s perpetual inventory method is commonly used for measuring capital stock: K_it_ = I_it_+(1-δ)K_it_−1, in which K indicates the capital stock; I, δ, and t mean the total fixed capital investment, depreciation rate, and year, respectively [49]. Referring to the relevant literature [50], the total fixed capital investment in the base period was divided by 10% as the base period capital stock, and the annual depreciation rate was 9.6% [49]. Outputs are classified into desirable output and undesired output. Concerning the relevant literature [45], GDP is selected as the desirable output; moreover, wastewater discharge, solid waste discharge, and an annual average value of PM2.5 in each region are set as the undesirable output. Here is a point to explain why the commonly selected industrial sulfur dioxide or waste gas is not selected. Because PM2.5 is currently an essential comprehensive indicator for the regional environment and air quality [51]. This paper uses the 2000–2019 NASA atmospheric environment remote sensing images to calculate the annual average concentration of PM2.5 to reflect the changes in air pollution in Zhejiang Province in a relatively accurate and long-term series.

**Table 2:** Ecological efficiency index system.

Properties	First-level index	Second-level index	Description of indicators	Unit
r	water	Water consumption	Water consumption of the whole society	10^4^ m^3^
	energy	Energy consumption	Electricity consumption of the whole society	10^4^ TCEs
	labour	Labour force	The number of urban employees	10^4^ persons
	land	Construction land	Built-up area	km^2^
	Capital	Capital stock	Total investment in fixed assets	10^8^ CNY
tsrntyindicators	desired output	Regional GDP	GDP	10^8^ CNY
	undesired output	Exhaust gas emission	The annual average value of PM2.5	μg/m^3^
		Wastewater discharge	Total wastewater discharge	10^4^ T
		Solid waste discharge	Total solid waste discharge	10^4^ T

### 3.4 Data source

The data about Zhejiang is mainly taken from *EPS*DATA (epsnet.com.cn) (2022-03-01) and Zhejiang Statistical Yearbook (http://tjj.zj.gov.cn/col/col1525563/index.html). The map comes from the Resource and Environment Science and Data Center (www.resdc.cn).

### 3.5 Methods

#### 3.5.1 Entropy method

To avoid the influence of artificial subjective scoring on the comprehensive development level of new urbanization, referring to the existing literature [1,52], this paper adopts the improved entropy method to evaluate the new urbanization value in each county. The calculation process has been fully illustrated in much existing literature.

#### 3.5.2 Super-SBM

The existing research shows that researchers have done much research on methods for calculating ecological efficiency, such as material flow analysis [53], ecological footprint [54,55], and DEA [49,56]. Among these methods, DEA, an essential approach for efficiency research[49,57], has been widely used, is considered a more scientific way to evaluate ecology, and has shown good reliability [58].

There are many advantages of using DEA. The main concept behind DEA is to keep the input and output indicators constant and establish a relatively efficient production frontier. The effectiveness of each decision unit is evaluated by comparing how much they deviate from the production frontier. This helps determine the true impact of each unit’s decisions. The effectiveness of DEA refers to the situation where the decision-making unit (DMU) falls on the production frontier [45]. DEA does not require a specific structure, nor does it require specific information. The evaluation process can objectively reflect the problem without being affected by human control. Furthermore, it can reflect the correlation between various element inputs. When evaluating the efficiency of a decision-making unit, the traditional DEA model can have an estimated value between 0 and 1, where 1 indicates an effective decision-making unit. Tone [59] proposed the non-radial and non-angular SBM model, directly incorporating the slack variables into the objective function. It can effectively solve the slack problem of inputs and outputs and ensure the accuracy of evaluation results after considering the unexpected outcomes. To solve the problem that [0,1] cannot be sorted under the premise of effective efficiency, a Super-SBM model [60] is proposed and widely applied. Therefore, this method will also be used to measure ecological efficiency in this paper.

#### 3.5.3 Coupling coordination degree model

The coupling degree model is a typical application of hard science principles in the field of soft science. Because of its precise meaning and simple operation, this model has been widely used in geographical research [61]. Coupling mainly refers to the phenomenon in which two or more systems affect each other through various interactive mechanisms. Drawing lessons from the related models of the coupling coordination degree in physics, the generalized calculation formula of the coupling degree can be expressed as Formula (1):

$$C(U_{1},U_{2},\ldots ,U_{n})=2\times {\left[\frac{U_{1}U_{2}\ldots U_{n}}{\prod_{i<j}(U_{i}+U_{j}{)}^{\frac{2}{n-1}}}\right]}^{\frac{1}{n}}$$ (1)

$$D=\sqrt{CT},T=\alpha_{1}U_{1}+\alpha_{2}U_{2}+\cdots +\alpha_{n}U_{n}$$ (2)

*C* refers to the coupling degree, *D* represents the coupling coordination degree, *T* stands for the comprehensive coordination index, *α*_*1*_, *α*_*2*_, …, *α*_*n*_ are the undetermined coefficients. Referring to the existing research[62–66] and combined it with the practical reality, this paper takes *α*_*1*_ *= α*_*2*_ *=* ,…, *= α*_*n*_ *= 1/n*.

So far, there have been no uniform classification criteria for the coordination degree. Based on the existing research [10,20], the coordination degree is categorized into five levels, as shown in Table 3.

**Table 3:** Classification criteria for coordination.

Coordination interval	Coordination level	Symbol	
0.0≤ D <0.3	General disorder		D1
0.3≤ D <0.4	Preliminary disorder		D2
0.4≤ D <0.5	Preliminary coordination		D3
0.5≤ D <0.7	Moderate coordination		D4
0.7≤ D <1.0	Quality coordination		D5

#### 3.5.4 Markov chains

Andrei Andreyevich Markov put forward the theory of Markov chains in his paper”Extension of the Limit Theorems of Probability Theory to a Sum of Variables Connected in a Chain” [67]. The Markov chain method is used to construct the Markov transition probability matrix and to characterize the spatiotemporal differentiation and evolution of the coupling coordination degrees in counties across different periods. First, the continuous coupling coordination degree is discretized into five types. Then, the probability distribution of the corresponding type and its interannual variation is calculated, approximating the evolution of the regional coupling coordination degree. If the probability distribution of the coupling coordination degree type in counties in year *t* is expressed as a *1 × k* state probability vector *F*_*t*_, denoted as *F*_*t*_ *= [F*_*1t*,_ *F*_*2t*,_*…*, *F*_*kt*_*]*, and the transition between the coupling coordination degree types in different years can be represented by a *k × k* Markov transition probability matrix *M*, the element *m*_*ij*_ represents the probability that an area belonging to type *i* in year *t* will be transferred to type *j* in the next year, and the following formula can calculate it:

$$m_{ij}=\frac{n_{ij}}{n_{i}}$$ (3)

In the formula, *n*_ij_ represents the sum of the areas that belong to type *i* in year *t* and are transferred to type *j* in year *t+1* during the study period, and *n*_I_ is the sum of the areas belonging to type *i* in all years. If the coupling coordination degree of a county in Zhejiang Province is *i* in the initial year and remains unchanged in the next year, the type transition of this region is defined as stable; if the coupling coordination degree of a county is improved, we define its type transition as upward transfer; Otherwise, downward transfer.

#### 3.5.5 Spatial autocorrelation analysis

The spatial weight matrix is the premise of spatial autocorrelation analysis and the basis of Moran’s I statistical test and model construction. *W*_*ij*_ stands for spatial weights, which are mainly categorized into spatial weights based on adjacency relationships and spatial weights based on distance relationships. Considering the theoretical and practical needs of the research [68], the minimum threshold distance of the spatial weights based on the distance relationship is chosen in this study.


$$W_{ij}=\{\begin{array}{ll} 1 & \;bound\;(i)\cap bound\;(j)\neq 0\\ 0 & \;bound\;(i)\cap bound\;(j)=0 \end{array}$$ (4)

Where *bound (i)* is the boundary of a spatial unit. It should be noted here that, considering that ZhoushanD, Shensi, and Daishan have no junction point on the map, according to the actual situation and common practice, we set the adjacency domains of Shoushan as NingboD and Daishan, the adjacencies of Daishan as ZhoushanD and Shensi, and the adjacency of Shensi as Daishan. Moran’s Index has been widely used to measure spatial correlation and has been recognized. Therefore, this paper will also use this index to measure spatial correlation.

#### 3.5.6 Geographical detector

Geographic detectors are a statistical method used to detect the spatial differentiation of geographic objects and reveal the driving factors behind them. They are widely used in many fields, such as economy and ecology [69,70]. This paper uses this method to explore and identify the differences in the influence of multi-dimensional factors on the coupling coordination degree. The specific formula is as follows:

$$q=1-\frac{\sum_{h=1}^{L}N_{h}\sigma_{h}^{2}}{N\sigma^{2}}$$ (5)

In the formula, *q* represents the explanatory ability of each influencing factor to the spatial differentiation of coupling coordination degree; The larger the value, the stronger the d ability; *h = 1*,⋯, *L* is the number of factor layers; *N*_*h*_ and *N* are the layer *h* and the number of samples in the entire region, respectively; *σ*_*h*_ and *σ* represent the variance of samples in the layer *h* and the whole area, respectively.

## 4 Results and analysis

### 4.1 Spatial and temporal evolution characteristics of new urbanization and ecological efficiency

**Fig 3:**
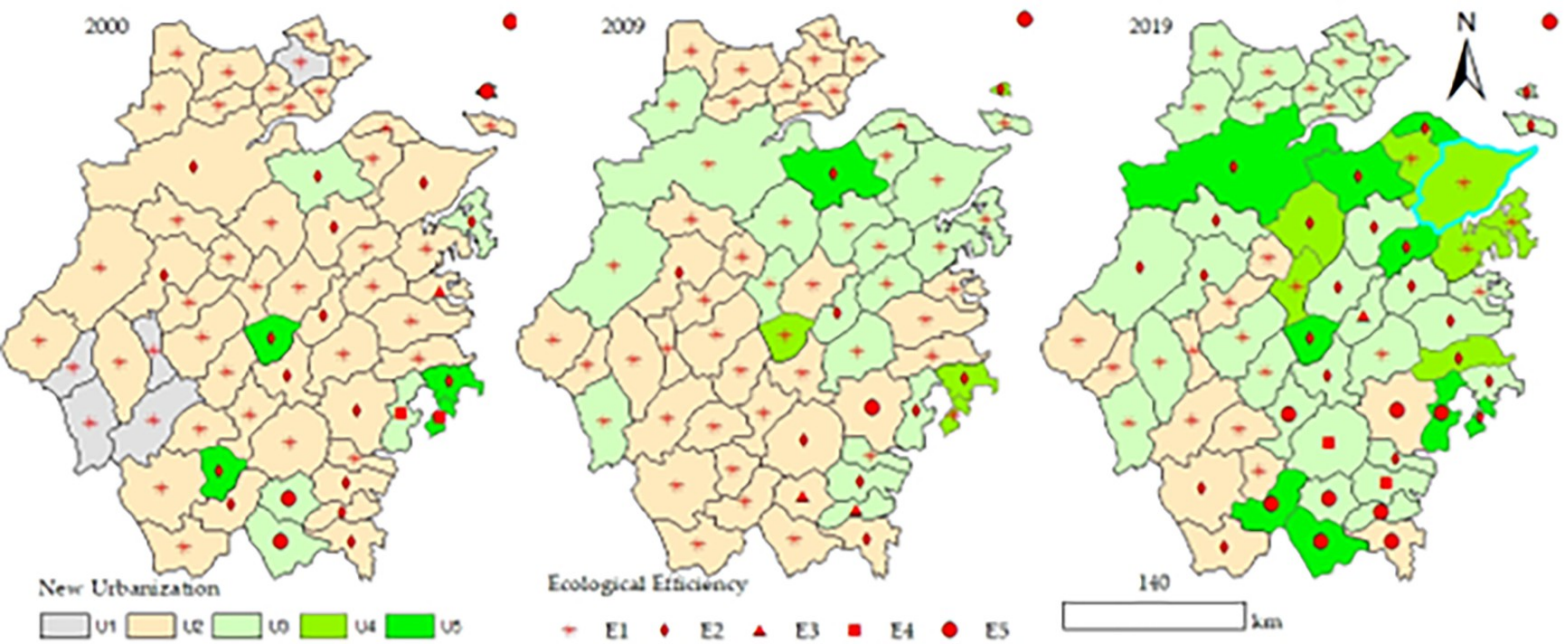
Spatial and temporal evolution of new urbanization and ecological efficiency. The map is obtained according to the map approval number: Zhejiang S(2022)34 https://zhejiang.tianditu.gov.cn/standard/view/842c093084084add9788a14a36536525, which meets the relevant provisions of the terms of service https://zhejiang.tianditu.gov.cn/about/clause.

Based on the panel data of 63 counties in Zhejiang Province from 2000 to 2019, the Super-SBM model is used to calculate the ecological efficiency (E) of each county in Zhejiang Province. According to the calculation results, the ecological efficiency value is divided into five grades, which are E1 (0.00 0.40], E2 (0.45 0.60], E3 (0.60 0.75], E4 (0.75 0.90], E5 (0.90 2.50]. Similarly, the entropy method is applied to measure the comprehensive development level of new urbanization (U) in each county in Zhejiang. The results are also divided into five categories, namely U1 (0.00 0.45], U2 (0.45 0.60], U3 (0.60 0.75], U4 (0.75 0.90], and U5 (0.90 2.50]. To more intuitively and clearly show their spatiotemporal evolution, 3-time interfaces of 2000, 2009, and 2019 are selected, and the grades and spatial distribution are visualized as shown in Fig 3.

#### 4.1.1 Spatial and temporal evolution of new urbanization

**Fig 4:**
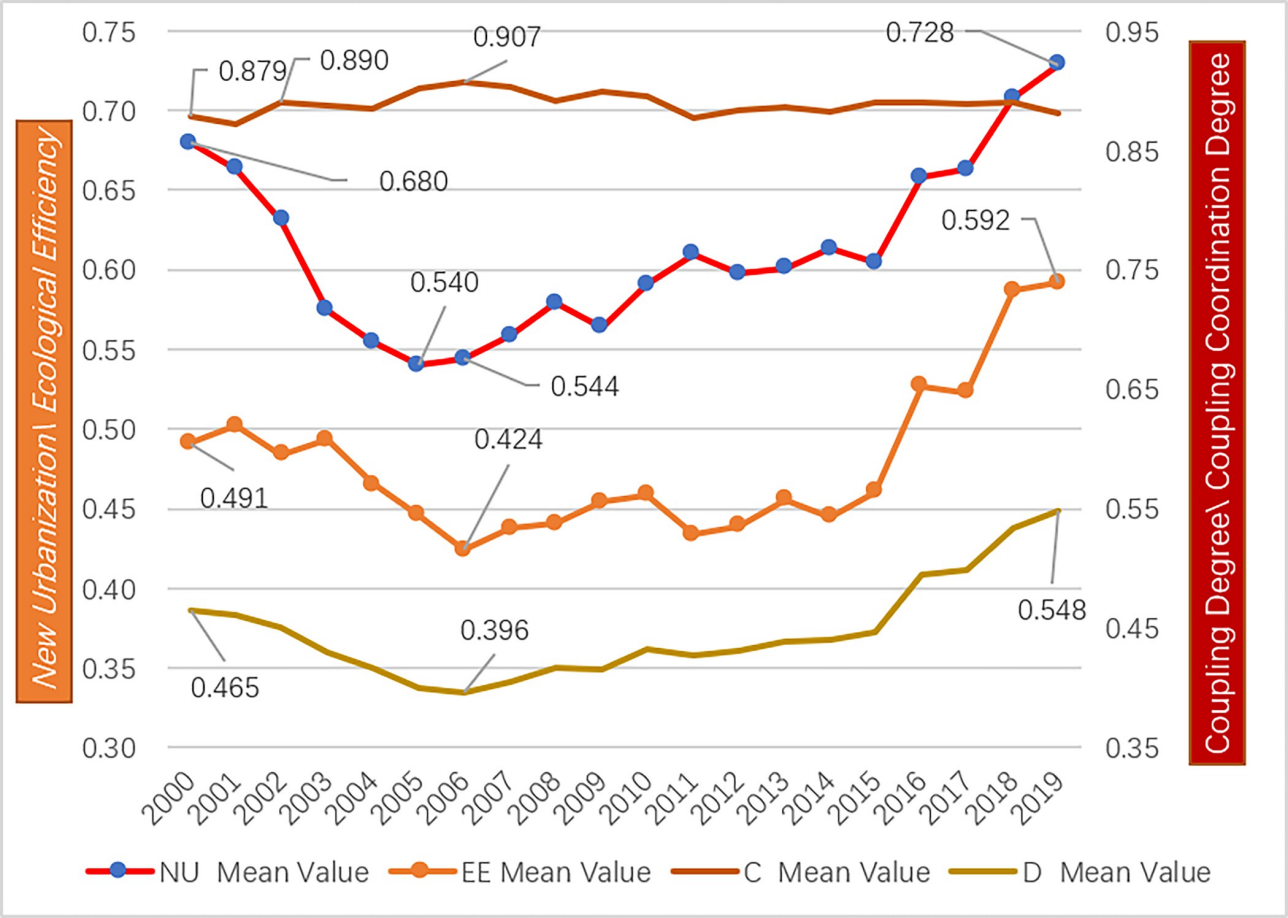
Development trends of new urbanization, ecological efficiency, coupling degree, coupling coordination degree.

**Fig 5:**
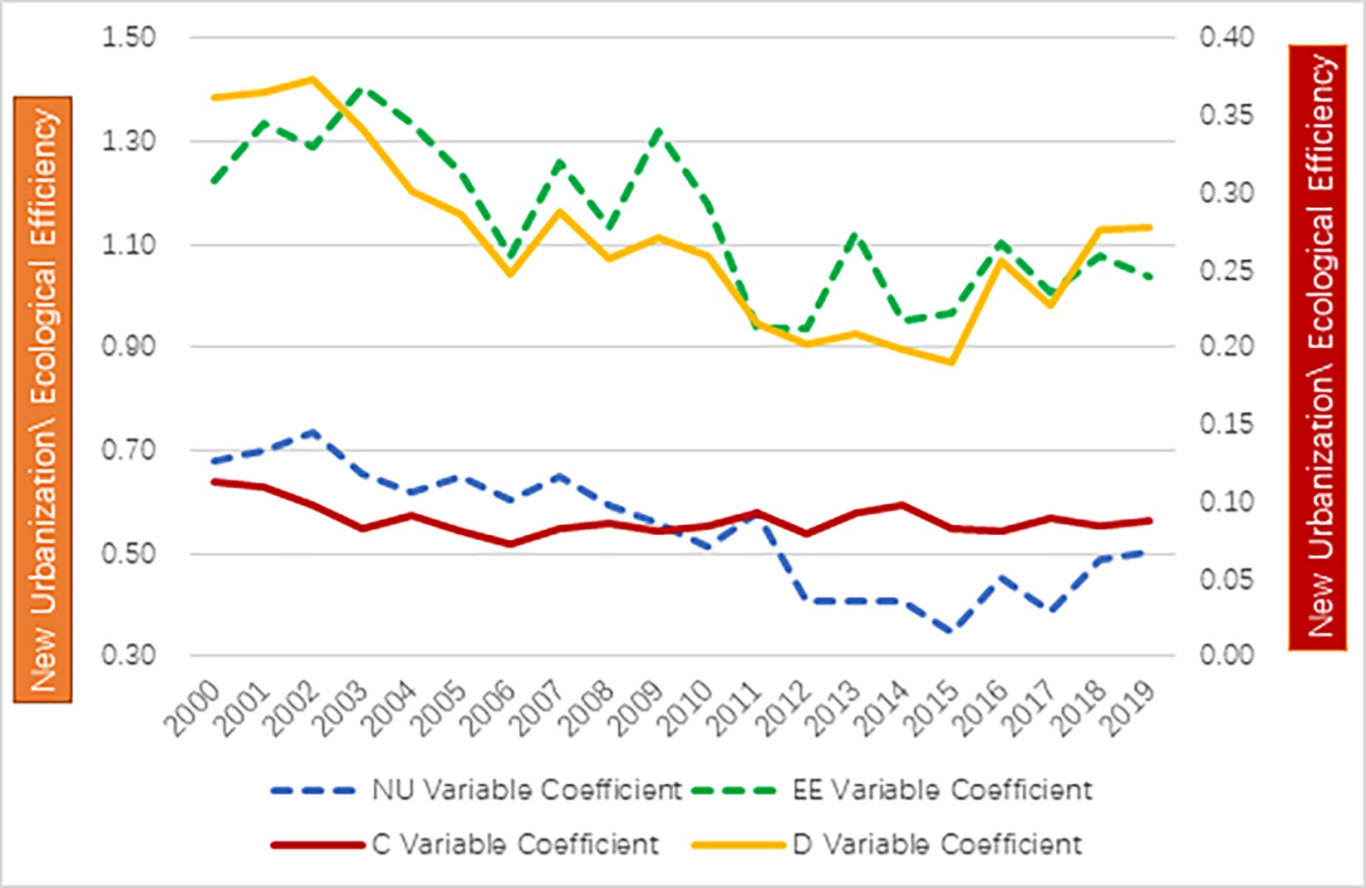
Development trends of coefficient of variation for new urbanization, ecological efficiency, coupling degree, and coupling coordination degree.

Fig 4 shows that the new urbanization has increased overall during the study period in Zhejiang Province, with an average value going up from 0.680 in 2000 to 0.728 in 2019. we can see that the change is relatively significant. The number of counties in the highest rank (U5) decreased to only one (Shaoxing) in 2009, then grew rapidly, reaching 9 counties in 2019. There are large fluctuations across cities. Taking Lishui City as an example, Lishui City (K) has jurisdiction over 6 counties, a county-level city (Longquan) and an autonomous county (Jingning). It is shown in Fig 3 that Yunhe County had gone through the most significant change, which was stabilized after 2009; Jingning County experienced the most increase, and Yunhe County had the most significant downturn. However, the average value of new urbanization at the city level is basically in line with that at the provincial level (Fig 4). Many previous studies conduct research at the provincial [42,71–73] or city level [6,20,74,75], which would result in losing specific and vital information regarding the actual situation of each county. Fig 5 shows that the overall urbanization gap between different counties is narrowing year by year. Compared with other indicators, the gap between counties is relatively tiny.

#### 4.1.2 Spatial and temporal evolution of ecological efficiency

Consistent with the existing research, the overall ecological efficiency of 63 counties shows a downward trend [76]. It reaches a certain minimum point, then increases, and offers an upward trend over the study period. Its average value declined from 0.491 in 2000 to the lowest point of 0.424 in 2006 and then increased rapidly in waves, reaching 0.527 in 2016 and 0.592 in 2019, an increase of 21% over the study period. Moreover, the ecological efficiency gap between counties tends to decrease year by year (Fig 5). Concretely, compared with the initial level in 2000, 11 counties, including Daishan County, have declined by 2019; 3 counties (Shengsi County, Wencheng County, and Taishun County) remain unchanged because they are always relatively optimal; the remaining 49 counties have increased in varying degrees, which are quite different among counties, cities and municipal districts. Among them, the fastest-growing area is the urban area of Wenzhou, which has increased by more than eight times; except for Xianju County and Jinyun County, all other counties have increased by more than 5%; the counties that jump four levels (E1-E5) are Lishui City and Jingning, and the counties that jump three levels (E2-E5) are Yongjia and Pingyang. The staged characteristic of “first decline and then rise” in ecological efficiency [77] has been verified. The average ecological efficiency of the base period was exceeded in 2016. However, the overall development trend should not obscure the specific conditions of the counties, and the corresponding measurement must be taken to meet the particular conditions of each county, especially the ones with declining ecological efficiency.

### 4.2 Dynamic evolution of coupling coordination degree

#### 4.2.1 Development trends of coupling degree and coupling coordination degree

From a macro view, the coupling degree between new urbanization and ecological performance maintains a high level, showing a wave-like development trend in an overall stable state. Its closing value is higher than the initial value. Specifically, Fig 4 demonstrates that the coupling degree in 2000 was 0.879, the first peak was 0.890 in 2002, and the second peak stood at 0.907 in 2006, the highest point in the entire study period. After reaching its lowest point in 2011, it remained steady. This phenomenon may be because the comprehensive efficiency value of the two systems has apparent fluctuations and a time gap. It is also related to the forerunner of new urbanization and ecological performance’s lagged and staged characteristics. In addition, during the 20-year study period from 2000 to 2019, it increased by 2% with an amplitude of less than 4.1%, which indicates the continuity and stability of the internal interaction mechanism of new urbanization and ecological efficiency.

From Fig 4, it can be found that the mean value of the coupling coordination degree between new urbanization and ecological efficiency presents a "U" shape development trend with an amplitude of 34.5%, which is 8.5 times that of the coupling degree. Its ending value is 1.2 times the beginning value over the study period. It hit the lowest point of 0.396 in 2006. When the two systems diverge, the coupling degree that measures their internal force will be enhanced. The coupling coordination degree reaches the lowest point, while the coupling degree reaches the maximum value. Thus, a relatively stable internal mechanism between new urbanization and ecological efficiency may exist.

#### 4.2.2 The variation of coupling coordination degree

The coefficient of variation is used to reveal the variation in coupling coordination degree among 63 counties in Zhejiang. The coefficient of variation can objectively reflect the degree of difference within a data set. It accurately reflects the data dispersion degree Compared to range, variance, and standard deviation. The smaller the coefficient of variation, the smaller the difference among counties in Zhejiang Province, and vice versa. Referring to relevant formulas, the coefficient of variation for each year is calculated as shown in Fig 5.

In Fig 5., we can see that the coefficient of variation of the coupling coordination degree in counties in Zhejiang Province has large fluctuations in the study period. It started increasing in 2000, peaked in 2002, then declined and hit bottom in 2015, after which it recovered somewhat but was still below the mean value for the entire study period. It has increased since 2015, so the dispersion among counties has become more extensive. Thereby, under the overall development trend, we must consider the specific situation of each county rather than directly adopt research conclusions based on prefecture-level cities or provinces.

#### 4.2.3 The Markov chain analysis of coupling coordination degree

Markov chain is a random process with discrete time and state. It can discretize continuous attribute values at different times, divide them into 5 types according to the value, and then calculate the probability distribution of each type and its change over time, which approximately shows the whole evolutionary process of the subject [78]. According to the classification criteria in Table 3, the transition probability of coupling coordination degree in 63 counties for different periods (with 2009 as the boundary) is calculated by the Markov chain, and the Markov transition matrix (see Table 4) is obtained as follows.

**Table 4:** Markov chain transition matrix.

Time	Type	D1	D2	D3	D4	D5
2000–2009						
	D1	0.00	0.67	0.33	0.00	0.00
	D2	0.04	0.76	0.20	0.00	0.00
	D3	0.00	0.53	0.40	0.07	0.00
	D4	0.00	0.31	0.46	0.23	0.00
	D5	0.00	0.00	0.14	0.57	0.43
2010–2019						
	D2	0.00	0.09	0.72	0.15	0.04
	D3	0.00	0.00	0.26	0.40	0.34
	D4	0.00	0.00	0.00	0.40	0.60
	D5	0.00	0.00	0.00	0.00	1.00

Table 4 displays that the evolution of the coupling coordination degree has prominent stage characteristics. It mainly transforms into a higher type or maintains the original style, and the leapfrog decline only appeared from 2000 to 2009. Specifically, in 2000, out of 63 counties studied, 3 counties were in the General Disorder (D1), 2 of which were turned into the Preliminary Disorder (D2); there were 25 counties in the Preliminary Disorder (D2), of which 1 county was turned into the General Disorder (D1), 76% stayed in the Preliminary Disorder (D2), and 20% was evolved to a higher level (D3); there were 15 counties in the Preliminary Coordination (D3), among which 8 counties retreated to the Preliminary Disorder (D2), 40% remained in the original type (D3), and 7% advanced to the upper level (D4); there were 13 counties in the Moderate Coordination (D4), 54% of which were dropped to the Preliminary disorder (D2), half of the remaining were kept in the original type (D4), and the other half entered the Quality Coordination (D5); there were 7 counties in the Quality Coordination (D5), of which 1 fell into the Preliminary Coordination (D3), 3 retreated to the Moderate Coordination (D4), and 3 maintained the Quality Coordination (D5). From 2010 to 2019, whatever the initial state, most counties evolved to a higher level, and several counties maintained the original type or leapfrogged up to a higher level. Moreover, the principal diagonal at different stages and its adjacent left and right sides have changed dramatically from 2000 to 2009. The sum of the probability figures at the lower left of the diagonal was more significant than that at the upper right, indicating that the coupling coordination degree between the new urbanization and the ecological efficiency in these 63 counties had a sign of recession. However, from 2010 to 2019, the situation was reversed. They were all distributed on the principal diagonal or its upper right with a noticeable improvement trend, which was in line with the evolution law of coupling coordination: first declining and then rising.

#### 4.2.4 Spatial and temporal evolution characteristics of coupling coordination degree

According to Table 3, the coupling coordination degree can be divided into five categories: General Disorder (D1), Preliminary Disorder (D2), Preliminary Coordination (D3), Moderate Coordination (D4), and Quality Coordination (D5).

**Fig 6:**
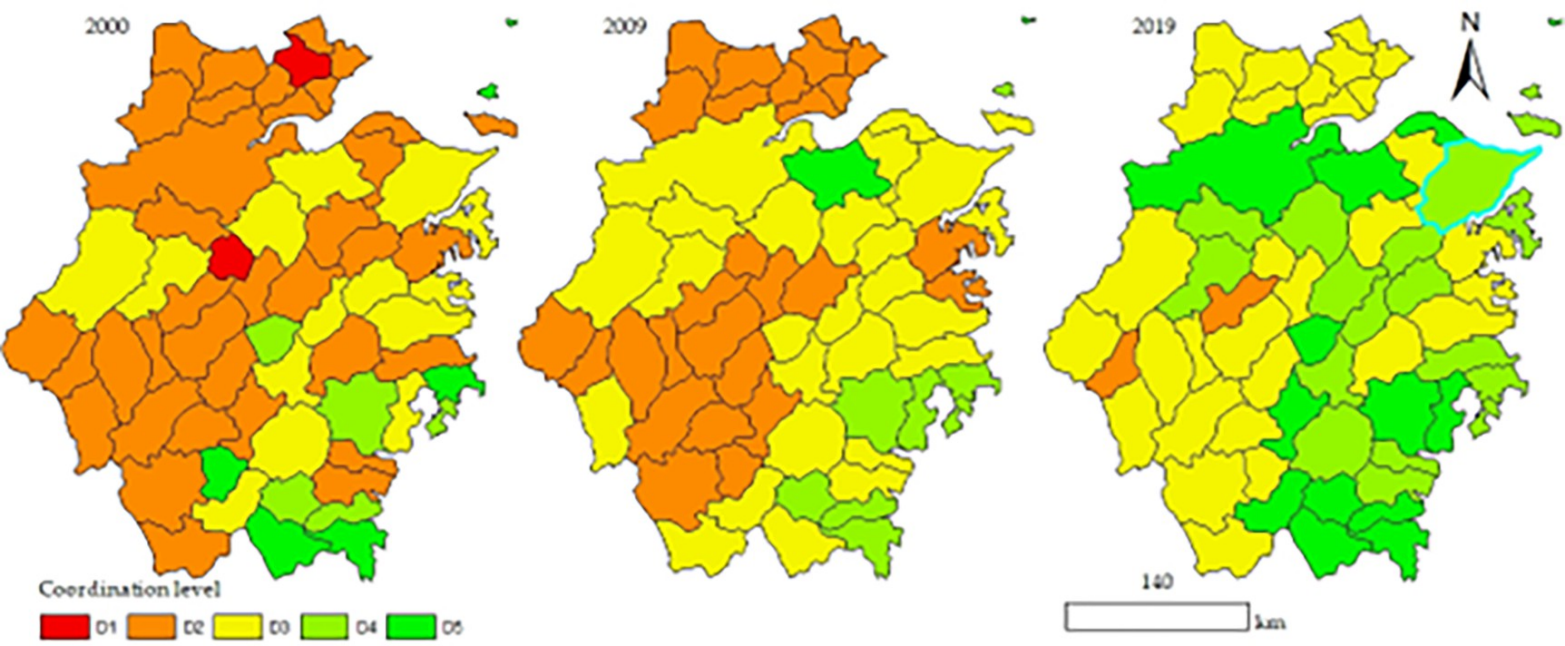
The spatiotemporal development characteristics of coupling coordination degree in counties in Zhejiang Province. The map is obtained according to the map approval number: Zhejiang S(2022)34 https://zhejiang.tianditu.gov.cn/standard/view/842c093084084add9788a14a36536525, which meets the relevant provisions of the terms of service https://zhejiang.tianditu.gov.cn/about/clause.

Fig 6 presents a vector diagram of the development characteristics of the coupling coordination degree in 2000, 2009, and 2019. We can see from the figure that the number of counties in the Quality Coordination (D5) increased from 6 in 2000 to 13 in 2019, the Moderate Coordination (D4) counties rose from 5 to 18, and the Preliminary Coordination (D3) counties increased from 14 to 30. At the same time, the number of counties in the General Disorder (D1) and Preliminary Disorder (D2) dropped from the original 2 and 36 in 2000 to 0 and 2 in 2019, respectively. In terms of specific counties, Daishan City went from Preliminary Coordination (D5) in 2000 to Preliminary Disorder (D4) in 2019, ending with a drop by one level throughout the study period. This may be because Daishan City has focused on economic development in recent years but neglected the environment. In addition, it is due to the multiple effects of national development policies. As a result, it has promoted the new urbanization but seriously weakened the ecological performance (see Fig 3), eventually dragging down the coupling coordination degree. Each research area has its development characteristics. We should conduct an analysis and judge according to each county’s actual situation.

#### 4.2.5 Spatial and temporal correlation characteristics of coupling coordination degree

**Fig 7:**
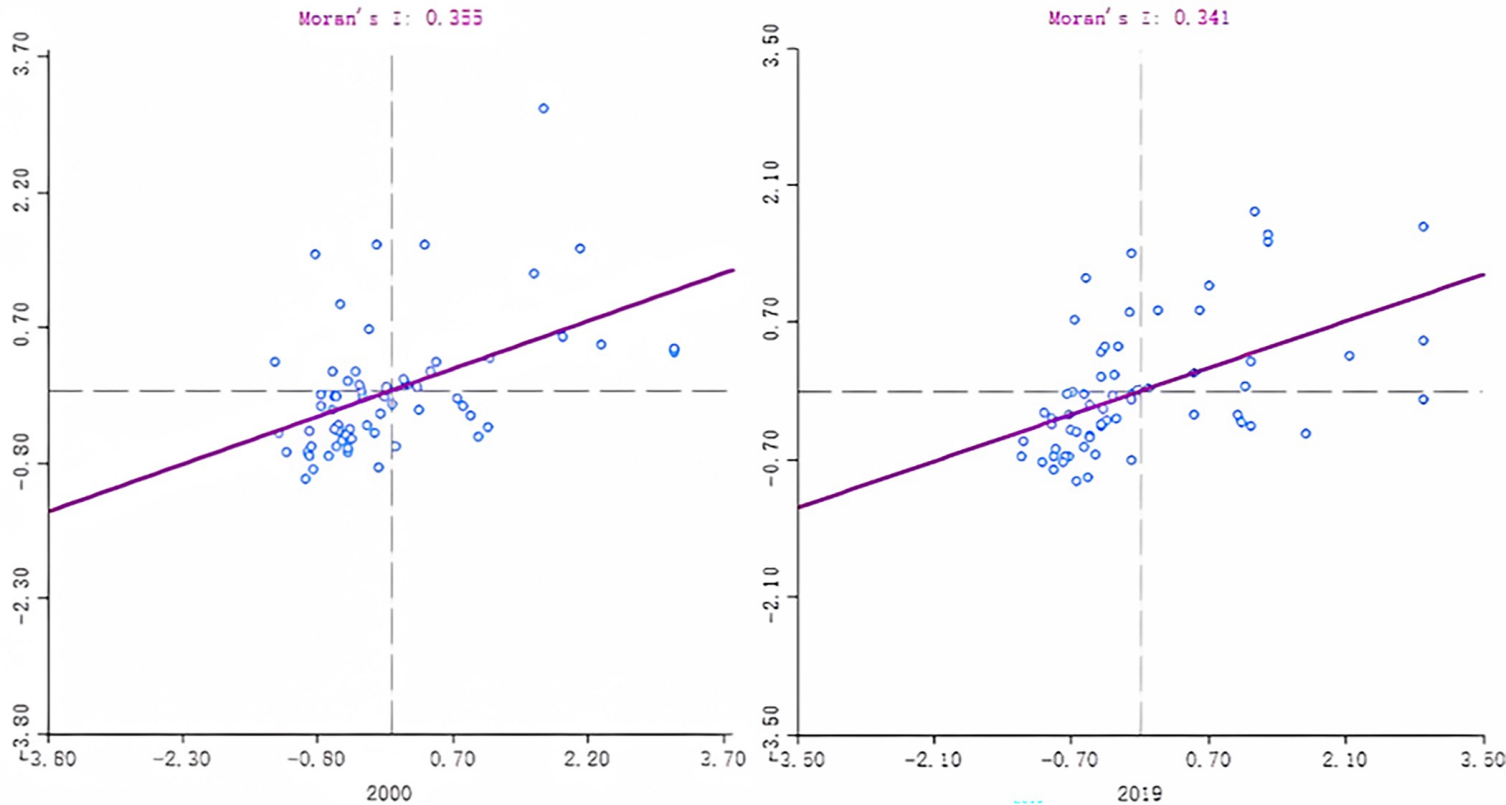
Moran’s I scatter plot for the coupling coordination degree in counties in Zhejiang Province in 2000 and 2019.

Moran’s I is a statistic for testing spatial autocorrelation, reflecting the degree of correlation among various counties in Zhejiang and their spatial distribution patterns. Its value is between [–1, 1]; less than 0 means negative correlation and spatial dispersion; more significant than 0 means positive correlation, and there is a spatial aggregation [6]. Based on the coupling coordination degree for each county in Zhejiang Province in 2000 and 2019, Moran’s I is calculated with the adjacency matrix (*Wij*). The results are shown in Fig 7. The Moran index is positive (0.355–0.341), indicating that the coupling coordination degree between the new urbanization and the ecological efficiency in counties in Zhejiang Province has a positive spatial correlation, and spatial agglomeration exists.

**Fig 8:**
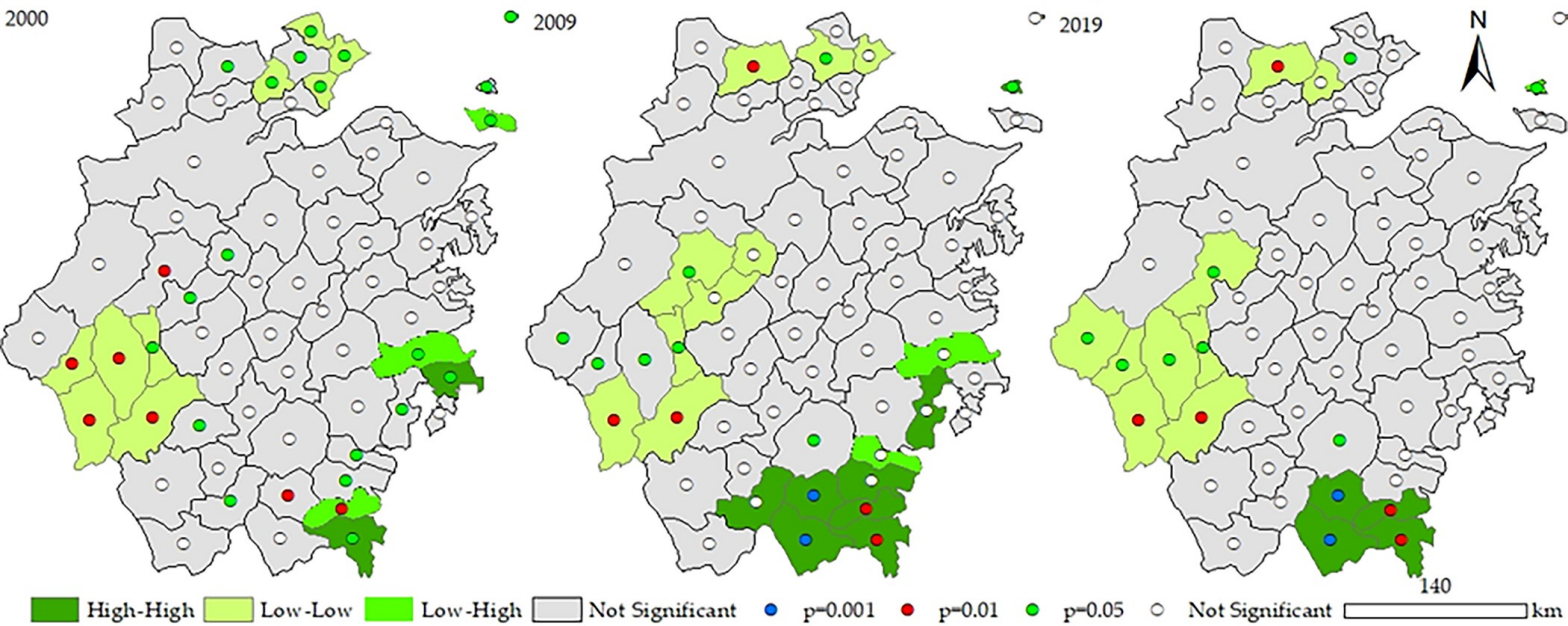
Local spatial correlation map of the counties in Zhejiang Province in 2000 and 2019. The map is obtained according to the map approval number: Zhejiang S(2022)34 https://zhejiang.tianditu.gov.cn/standard/view/842c093084084add9788a14a36536525, which meets the relevant provisions of the terms of service https://zhejiang.tianditu.gov.cn/about/clause.

The distribution situation can be noticed from the scatter diagram in Fig 7. and the local spatial correlation diagram in Fig 8. That is, the Low-Low type (the third quadrant) and the High-High type (the first quadrant) dominate, which is followed by the Low-High (second quadrant) and the High-Low type (the fourth quadrant). In 2000, there were 13 districts in the first quadrant, 3 of which passed the significance test, namely Shengsi, Wenling, and Cangnan; there were 7 districts in the second quadrant, 3 of which passed the significance test, namely Zhoushan City, Taizhou and Pingyang; the third quadrant has 36 districts, 9 of which passed the significance test and was distributed in Jiaxing, Quzhou and Suichang; the fourth quadrant has 7 districts, among which Jiande passed the significance test. In 2019, the agglomeration phenomenon became more apparent. Except for Daishan, the rest of the districts that passed the significance test were all in the H-H and L-L types, and the quantity either increased or decreased. It is also worth noting that Jiande has entered the L-L type from the original H-L type. Initially, the neighboring districts were relatively low in the coupling coordination degree. At the same time, Jiande developed well, and its coupling coordination degree was higher than its neighboring districts, which had a positive pulling effect on its neighboring districts. Reversely, the neighboring low-value districts harmed it.

## 5. Factors influencing the coupling coordination degree between new urbanization and ecological efficiency in Zhejiang counties

### 5.1 Variable selection

To further explore the influence of county-level spatial heterogeneity on the coupling coordination degree between new urbanization and ecological efficiency in Zhejiang Province, referring to the research results of related scholars [79], this paper selects 12 factors in three dimensions: nature, economy, and society. With the help of ArcGIS’s natural breakpoint method, each aspect is discretized by quintile. GeoDetector is used to detect the influence of each factor on the coupling coordination degree between the two and their interaction. Specifically, in terms of natural resources, four variables are involved. They are the annual precipitation (Z1) that reflects and decides regional water resources and is one of the critical factors affecting environmental quality; the sunshine hours (Z2) have a significant impact on vegetation distribution and urban ecology and indirectly affect air quality; the average temperature (Z3) that affects human survival, production, living, and biological distribution; the population density (Z4) that reflects environmental carrying capacity and urban population agglomeration. In terms of economic development, we select the level of opening to the outside world (J1) that reflects the openness and inclusiveness of the economy, regional industrial structure (J2), regional industrialization level and development stage (J3), and regional innovation capability (J4) as variables. At the social level, the annual consumption per capita (S1) reflects the overall living cost and social consumption level in regions, the government support (S2), the informatization level (replaced by the Internet development level (S3)), and the People’s educational level (S4) that reflects people’s education degree are selected as variables as shown in Table 5.

**Table 5:** Variables selected for analysis.

Category	Factor	Indicator	Unit	Code
natural resources	water resource environment	annual precipitation	mm/year	Z1
	Solar radiation	sunshine hours	hours/year	Z2
	temperature condition	average temperature	°C	Z3
	environmental carrying capacity	Population density	people/km^2^	Z4
economic development	degree of openness	The ratio of the sum of imports and exports to GDP	%	J1
	Industrial structure	The ratio of tertiary industry output to GDP	%	J2
	Industrialization level	The ratio of industrial output to GDP	%	J3
	Innovation capacity	The number of patent licenses per 10,000 person	pieces/10^4^person	J4
social conditions	social consumption level	Annual social consumption per capita	10^4^ CNY/ person	S1
	government support	General budget expenditure as a percentage of fiscal revenue	%	S2
	Internet development level	The number of Internet users/permanent residents	households/10^4^ persons	S3
	People’s educational level	Average years of education*	years	S4

*Note: Average years of education = (6×P primary school+9×P junior high school+12×P high school+16×P college or above) / (P primary school + P junior high school + P high school + P college or above), where P means a person.

### 5.2 Explanatory power of the influencing factors

Table 6 shows the *q* value of each dominant factor and its interaction with geographic detectors from 2000 to 2019. In general, the differences in economy, society, and nature are the three major driving forces that cause the heterogeneity of the coupling coordination degree in each country.

**Table 6:** The *q* value of factors and factor interaction.

Detection factor	Z1	Z2	Z3	Z4	S1	S2	S3	S4	J1	J2	J3	J4
Z1	0.093											
Z2	**0.165**	0.047										
Z3	0.166	0.119	0.079									
Z4	**0.181**	**0.119**	**0.162**	0.066								
S1	0.127	0.102	**0.145**	**0.132**	0.056							
S2	0.136	0.119	0.147	**0.186**	**0.181**	0.114						
S3	0.112	**0.086**	**0.115**	**0.100**	**0.108**	**0.162**	0.031					
S4	0.122	0.118	0.150	**0.154**	0.094	**0.192**	**0.124**	0.076				
J1	**0.139**	**0.095**	0.117	**0.114**	**0.098**	0.119	**0.093**	**0.121**	0.041			
J2	0.210	**0.181**	0.211	**0.203**	**0.195**	0.197	**0.171**	**0.207**	**0.187**	0.133		
J3	0.300	0.267	**0.310**	**0.327**	**0.291**	0.271	**0.281**	0.282	**0.270**	0.309	0.223	
J4	0.100	**0.089**	**0.121**	**0.091**	**0.082**	**0.173**	**0.066**	**0.113**	**0.071**	**0.169**	**0.282**	0.023

**Note:** The value in bold means that two factors produce a nonlinear enhancement effect after the interaction.

Economic development is the key. The explanatory power of the industrialization level is the highest (0.223), followed by the explanatory power of the industrial structure. At the same time, the degree of openness and innovation capability have a relatively low impact compared with other factors. Industrialization is an essential direction toward development and the focus of transformation in developing new urbanization. It transforms the production mode of enterprises and realizes the change and upgrading them, thereby improving the economic green effect and the ecological efficiency in the regions. On the one hand, the level of industrialization will generally affect the discharge of industrial wastewater, the emission of industrial waste gas, industrial smoke, and dust in the region, and the consumption of resources. In addition, it will also harm the environmental quality of the entire area, especially the PM2.5 concentration, an important indicator that reflects the air quality. On the other hand, industrialization development can beneficially promote the regional economy, improve living standards, and financially support all aspects. The two-way effect of industrialization has played a significant role in the coupling coordination between the new urbanization and ecological efficiency. The optimized layout of the industrial structure, especially the development of high-tech industries such as the tertiary industry and the service industry, through the spillover and diffusion of new technologies and the in-depth development of the service industry, will significantly promote the new urbanization and the ecological performance, thereby affecting the coupling coordination between them.

Natural conditions are the foundation. Annual rainfall can explain 9.3% of the difference in the coupling coordination degree. Water is the source of life and significantly impacts human production and energy and the survival of animals and plants. Water has played a non-negligible role in forming regions and economic development since ancient times. Therefore, it is the most important influencing factor in natural conditions. As the premise and foundation for forming the population gathering areas, water resources have certain impacts on economic agglomeration and industrial and agricultural production. It interacts with the population density to form an explanatory power of 18.1% for the coupling coordination degree. Sunshine hours are the weakest driving factor in natural conditions, and it dramatically correlates with the distribution of regional greenery and natural vegetation, thus affecting the regional PM2.5 and the environmental quality. It has little impact on the new urbanization and, therefore, has little explanatory power over the coupling coordination degree. The average temperature and temperature conditions affect the new urbanization through labor productivity, energy conservation, and emission reduction. At the same time, they also indirectly impact ecological performance in the same way. Therefore, as an essential part of the natural resource category, its explanatory power is second only to the water factor, and its interaction with population density is also nonlinearly enhanced.

Social civilization is another important motivator. Government support is important in guiding and guaranteeing, so it ranks third. Guiding and supporting the transformation and upgrading of relevant industries is conducive to improving the new urbanization and the environment in counties and is also beneficial to developing the regions towards a high-quality level. The explanatory power of per capita savings on the coupling coordination degree reaches 6.9% among all the social factors. Rescues have a positive effect on economic growth [80]. The increase in household savings provides financial support for transforming and upgrading industry and agriculture and expanding production in society. It provides the necessary financial guarantee for promoting the new urbanization in energy conservation and emission reduction, medical care, and education. It acts as a reservoir through intermediaries such as banks.

Regarding the joint effect of different factors, 43 out of 66 interaction groups produced a "1+1>2" nonlinear enhancement effect. For example, innovation capability has a single-factor impact of only 2.3%, but its interaction with other variables (up to 28.2%) is a nonlinear enhancement. As an exception, it is a two-factor enhancement (10%) with annual rainfall. Therefore, its influence on the coupling coordination degree between new urbanization and ecological efficiency should not be ignored. The remaining 23 groups of interaction factors are in the two-factor enhancement segment, which further proves that the coupling coordination degree between new urbanization and ecological performance is the result of the combined effects of various natural, social, and economic factors and the explanatory power of economic development > the explanatory power of natural conditions > the explanatory power of social. It should not be viewed one-sidedly from the perspective of a single factor, and the interaction between factors cannot be ignored.

## 6 Discussion

### 6.1 How to construct the new urbanization and the ecological efficiency index systems?

Both academic and practical circles believe that it is not scientific enough to rely solely on the non-agricultural population to measure the level of urbanization. The official urbanization level is now generally measured by the permanent resident population. It cannot reflect the natural process of urbanization in China. From 2000 to 2019, measured from a population perspective, it increased by 21.3%, while with the new urbanization measurement in this paper, it rose by only 10%. The official announcement is that urbanization has been increasing throughout the whole process of its development. However, judging from the data studied in this paper, it developed in a "U" shape. It reached its lowest point (provincial average) in 2005, similar to the results from many other measurements using comprehensive indicators to evaluate urbanization [81]. Therefore, total indicators must be adopted because various aspects are involved in urbanization, especially the "high growth, high pollution" at the beginning of this century [1]. It is necessary to establish an urbanization process evaluation system that is more objectively aligned with sustainable development and suitable for developing countries like China. Referring to the relevant literature [44,45,71], this paper constructs a "five-in-one" evaluation index system for the new urbanization, especially in cultural aspects. Although the role of culture is imperceptible, its influence cannot be underestimated and must be paid attention to. Therefore, this paper has made innovations in this aspect to show better the actual situation of urbanization in various counties in Zhejiang Province.

Similarly, ecological efficiency indicates ecological development status and sustainability. Further in-depth analysis of its indicator system, including measurement methods, is significant. Today, China has been slowly freed from the era of high pollution and growth, which the treatment level of wastewater and solid pollutants can verify well. Air quality has become the protagonist, especially the exhaust gas generated by the consumption of fossil fuels, which has seriously affected the quality of the regional environment. For this reason, regarded as an undesired output of ecological efficiency, the concentration of PM2.5 in the regions is selected to measure the regional air quality more comprehensively and scientifically. This paper believes that when we measure ecological efficiency, the actual situation of the areas and the critical factors affecting the regional environment should be fully considered so that the scientific nature of ecological efficiency and the region’s sustainable development can be better reflected. Therefore, we should comprehensively consider and dynamically select indicators for ecological efficiency that reflect its real value to realize better the research’s starting point and purposes.

### 6.2 What affects the coupling coordination degree between new urbanization and ecological efficiency in Zhejiang counties?

Many studies have shown the coupling relationship between new urbanization and the ecological environment [1,6,44,45,82]. Aside from the traditional econometric model (including spatial metrology), this paper adopts a new perspective of the geographic detector to analyze the influencing factors of the coupling coordination based on regional heterogeneity. Regarding relevant theories and internal correlations, this paper measures the influencing factors with 12 variables in three categories: nature, society, and economy, thus drawing more valuable conclusions than ever. Nowadays, many studies focus more on economic development and worry about metering drawbacks like the collinearity among variables so that some important findings may be discarded. From Table 6, we can find that many factors affect the coupling coordination degree. Economic factors are still the mainstream, mainly reflected in industrialization and industrial structure. Therefore, the mode and format of economic development will significantly impact the coordinated development of regional urbanization and ecology. The economy is followed by government support in social factors and annual rainfall in natural factors. Overall, the influence on the coupling coordination degree ranks as economy>nature>society. The natural conditions in the regions cannot be ignored, especially the rainfall. A certain amount of rainfall is beneficial to the improvement of air quality, as well as the improvement of the ecological environment and the development of cities and towns. It is worth mentioning that although innovation benefits economic growth, its impact on the coupling coordination between the two systems is the weakest among all the influencing factors. This may be because innovation works through improving other terms, such as economy and society, and has been absorbed by other factors, making its direct effect less noticeable. In a word, when analyzing the development trend of the coupling coordination between the two systems, we should comprehensively evaluate the aspects of nature, economy, and society. In particular, we should always maintain the heterogeneity of physical and geographical conditions and analyze only the economy or other aspects.

### 6.3 Does the “Two Mountains” theory work?

On 15 August 2005, Xi Jinping went to Yucun village for an investigation. Bao Xinmin, the then secretary of the village party branch, reported that Yucun Village had adopted a democratic decision to shut down the mines that polluted the environment and started working. President Xi Jinping said: “You must stop thinking about going the old way and still being so obsessed with the old development model. So, as you said just now, it is a perspicacious move to make up your mind to close some mines. Lucid waters and lush mountains are invaluable assets. We used to say that we need lucid waters, lush mountains, and invaluable assets. Lucid waters and lush mountains are invaluable assets”. This is the first time President Xi Jinping has put forward the ecological and environmental protection concept of “lucid waters, and lush mountains are invaluable assets,” namely, the “Two Mountains” theory. Then, it explained the relationship between economic development and environmental protection from different points of view [83] and answered a series of essential questions, such as what ecological civilization is and how to build one. The two Mountains theory’s development context and substantive meaning reveal that the concept of ecological environmental protection is emphasized, which aligns with the essence of green economic development. Second, shutting down high-input, high-output, and high-polluting enterprises and developing tertiary industries is emphasized. It is a guideline to be followed in dealing with the relationship between economic development and environmental protection [57]. It does promote environmental protection and improves environmental quality. In Figs 3 and 4, we can see that ecological efficiency reached its lowest value in 2006, the second year after the theory was put forward, while new urbanization dropped to the bottom in the same year. After the policy was put forward, it immediately drew the attention of the whole province, which was first demonstrated by the new urbanization. Because of China’s unique national conditions, the influence of the country’s leaders is far beyond the general slogans and appeals made in foreign countries. Policies can be implemented quickly and efficiently as this will affect local officials’ performance and political careers [84,85]. From Table 6, We can also find that government support ranks third among all the influencing factors. Therefore, the “Two Mountains” theory can effectively promote the transformation of regional economic development mode and increase government support. At the same time, it can encourage regional ecological environment protection and the sustainable development of the economy, society, and environment, thereby enhancing the coordination between new urbanization and ecological efficiency.

## 7. Conclusion and policy implication

### 7.1 Conclusion

Through the above analysis and discussion, this paper draws the following conclusions:

1. The overall county-level new urbanization in Zhejiang Province has experienced a "U"-shaped wave-like development process that first decreased and then increased. Throughout the study period, it reached its lowest point in 2005, broadly consistent with the previous findings taking provincial and municipal levels as research samples. However, there are no apparent rules specific to each study unit (county-level), and the conclusions are not unified and vary greatly. In this regard, previous studies were largely conducted based on China’s provinces or prefecture-level cities with the conclusions being similar. In comparison, this paper is based on the county level with conclusions being quite different. Therefore, it is irrational to cover up the actual situation of smaller-scale regions with the findings obtained from larger-scale research.
2. The overall ecological efficiency of counties in Zhejiang Province has a "U" shape development process, which is a significant difference from previous findings based on provincial and prefecture-level cities (one-way up or down), similar to the new urbanization. But its specific development trend is different from that of new urbanization, with the lowest point appearing in 2006. From a single-county perspective, each county’s eco-efficiency development is different. The ecological efficiency in most counties has improved, and the gap between counties is gradually narrowing.
3. The coupling degree between new urbanization and ecological efficiency in the counties in Zhejiang Province remained unchanged. In contrast, the coupling coordination degree first decreased, reaching its lowest point in 2006, then rose in large waves mixed with small waves. The coefficient of variation for the county-level coupling coordination degree in Zhejiang Province generally showed a downward trend, but it rebounded after touching the lowest level in 2015. For the specific counties, the ups and downs are apparent. The number of counties in the General Disorder (D1), Preliminary Disorder (D2), and Quality Coordination (D5) stages in 2000 were 3, 25, and 7, respectively, and the corresponding numbers in 2019 were 0, 2, and 13. The mainstream is Preliminary Coordination (D3) (30 counties), accounting for nearly half. At the trough in 2006, however, 44 counties were in the Preliminary Disorder (D2) stage, far more than 25 at the beginning. The evolution of the coupling coordination degree at the county level in Zhejiang Province is generally dominated by the advancement toward the neighborhood level and the maintenance of the original type. Leapfrog growth is not apparent, and decline only occurs in the first half of the study period.
4. The coupling coordination degree between new urbanization and ecological efficiency has an excellent positive spatial correlation and shows apparent High-High and Low-Low agglomeration over time. As the absolute value of Moran’s I index decreases, the spatial dependence is strengthened.
5. For the driving power of coupling coordination degree, the explanatory power of economic development > the explanatory power of natural resources > the explanatory power of social conditions. The interaction of factors shows the nonlinear enhancement of "1+1>2" and the intrinsic complementary enhancement effect, which occupies 52 out of 66 groups. The interaction of rest factors is all two-factor enhancement. Although some aspects have weak explanatory power alone (such as innovation), they are the most critical interaction objects when they act together with other factors.

### 7.2 Policy recommendation

Combined with the above conclusions, the following policy recommendations are put forward to promote the coupling coordination degree between new urbanization and ecological efficiency:

1. The government should enforce the “Two Mountains” Theory further. Past practice has quantitatively shown that new urbanization and ecological efficiency in counties can develop side by side to achieve coordinated development. Therefore, this policy should be implemented firmly and consistently.
2. Pay attention to the significant role of industrialization, industrial structure, and government support in promoting the coordination between new urbanization and ecological efficiency. As the top three reasons for improving the coupling coordination, the counties in Zhejiang should further develop productivity, enhance industrialization, promote production efficiency, and pay attention to upgrading the economic structure and government support.
3. The government should adhere to the synergy and comprehensive promotion of various influencing factors. Whether it is social, natural, or economic factors, the characteristics of "non-linear enhancement" and "two-factor enhancement" with other factors should be fully considered.

This paper seeks a more microscopic county perspective to analyze the new urbanization and ecological efficiency. It introduces PM2.5 as a variable in the ecological efficiency evaluation system and adds the element of cultural urbanization. Using Super-SBM with unexpected output and entropy method, the ecological efficiency and the new urbanization of 63 counties in Zhejiang Province are calculated. The calculated efficiency value is a relative value rather than an absolute one. The subsequent county-level classification, evolution, Markov transfer chain, etc., are all conducted based on the relative importance, and their results are affected by the selection of regions, input-output variables and so on, to a certain extent. Therefore, comparative analysis or fine research by multi-methods in the different areas is also worth further discussion.

## Supporting information

S1 Data (XLSX)
